# Analysis of Intracellular Metabolites from Microorganisms: Quenching and Extraction Protocols

**DOI:** 10.3390/metabo7040053

**Published:** 2017-10-23

**Authors:** Farhana R. Pinu, Silas G. Villas-Boas, Raphael Aggio

**Affiliations:** 1 The New Zealand Institute for Plant & Food Research Limited, Private Bag 92169, Auckland 1142, New Zealand; 2 School of Biological Sciences, University of Auckland, Private Bag 92019, Auckland 1010, New Zealand; s.villas-boas@auckland.ac.nz; 3 Department of Cellular and Molecular Physiology, Institute of Translational Medicine, University of Liverpool, Crown Street, Liverpool L693BX, UK; raphael.aggio@gmail.com

**Keywords:** metabolite footprinting, exometabolome, culture medium, metabolic modelling, sample preparation, analytical instruments, data integration, extracellular metabolites, intracellular metabolites

## Abstract

Sample preparation is one of the most important steps in metabolome analysis. The challenges of determining microbial metabolome have been well discussed within the research community and many improvements have already been achieved in last decade. The analysis of intracellular metabolites is particularly challenging. Environmental perturbations may considerably affect microbial metabolism, which results in intracellular metabolites being rapidly degraded or metabolized by enzymatic reactions. Therefore, quenching or the complete stop of cell metabolism is a pre-requisite for accurate intracellular metabolite analysis. After quenching, metabolites need to be extracted from the intracellular compartment. The choice of the most suitable metabolite extraction method/s is another crucial step. The literature indicates that specific classes of metabolites are better extracted by different extraction protocols. In this review, we discuss the technical aspects and advancements of quenching and extraction of intracellular metabolite analysis from microbial cells.

## 1. Introduction

Metabolomics can be described as a group of techniques applied to detect, identify and potentially quantify small organic molecules (<1.5 kD) produced or modified by living cells [1]. These techniques have been successfully applied in a wide variety of fields, such as chemistry, engineering, medicine and biology [2,3,4,5]. In microbiology, metabolomics has been extensively applied to analyze the intra- and extracellular metabolites present in microbial samples [6,7,8]. 

Ideally, metabolomics techniques should represent the metabolic state of a microbial population at the exact moment that a sample is harvested and under the environmental conditions in which the cells were growing. However, many important metabolites involved in cell metabolism, such as ATP and NADH, can quickly be metabolized by enzymes or degraded (<1 mMs^−1^) when exposed to factors such as temperature and light [9]. Consequently, the level of these metabolites may change very rapidly during sampling and sample preparation, which would change their final concentrations, producing results that may not represent the population’s true metabolic state. To acquire accurate metabolomic results, the cell’s metabolism must be quickly stopped, or quenched, before or during sampling [10,11].

One key aim of metabolomics is to obtain as much information as possible about metabolite levels associated with a biological sample. Intracellular metabolites are contained within a mechanical barrier, the cell membrane or cell envelope. Therefore, in order to identify and quantify intracellular metabolites, it is necessary to extract metabolites from the intracellular compartment. This is usually achieved using extracting solvents (organic, inorganic non-aqueous or a mixture of the two) that make the cell′s envelope porous, or *permeable*, allowing the penetration of these solvents into the intracellular medium and greater recovery of intracellular metabolites. This process also helps to separate “small” metabolites from macromolecules such as proteins and nucleic acids. The complete disruption of the cell wall is unnecessary and on most occasions undesirable, as it would result in the release of both small and large molecules into the extraction solution [9] and metabolomics aims at analysing small molecules only.

An ideal method for extracting intracellular metabolites would be reproducible, able to equally release intracellular metabolites of different classes whilst preventing chemical and biochemical degradation and assuring the extraction goes to completion [12]. Completion cannot be easily verified as metabolite levels are usually unknown a priori. The performance of an extraction method can be assessed by applying different extraction methods to the same biological sample and then comparing the methods’ abilities to extract the different metabolites. The latter is referred to as the extraction *efficacy*. The level of metabolite degradation associated with an extraction method can also be assessed. This is carried out by spiking metabolite standards into biological samples and measuring the recovery rates of the different metabolites [13]. Losses during metabolite extraction can be corrected by using metabolite-specific recovery factors or by applying adequate internal standards (e.g., isotope-labeled compounds) [9,14]. The latter is recommended when possible. 

A complete metabolome analysis of microbial cells would involve the following steps:

(a) Growing the microorganism under study using an appropriate culture media;
(b) Sample collection at suitable or desired stage of growth and quenching of microbial cells;
  i Separation of microbial cells from growth media: The supernatant is used for extracellular metabolite analysis;
  ii Microbial cells are used for intracellular metabolite analysis;
(c) Extraction of intracellular metabolites;
(d) Metabolite analysis of intra- and extracellular metabolites using an appropriate instrumental approach.

## 2. Cell Metabolism and Metabolite Turnover

Chemical compounds are the energy sources and the building blocks of a cell. They are used to perform both essential biological functions and to maintain the structure of the cell. When a chemical compound is oxidized or converted into a different compound with lower free energy content, it releases energy that can be stored and used to perform biological processes. The set of chemical conversions occurring in the cell is referred to as metabolism, while the chemical compounds involved in metabolism are referred to as metabolites [1,15].

Conversion between metabolites, or turnover, is predominantly performed by enzymes, i.e., proteins that are able to reduce the energy required to modify the structure of a specific compound. Whether an enzyme actually converts one compound into another and how quickly this happens depends on a number of factors [16]. The substrates and cofactors required by each enzyme must be available at specific levels. High or low levels of certain metabolites can actually act as inhibitors to some enzymes (e.g., negative feedback). Activators may also be required and environmental factors, such as temperature and pH, also determine the rate of conversion performed by an enzyme. Metabolites can also spontaneously interact with each other or be degraded by factors such as temperature or light [9]. Therefore, the level of each metabolite inside the cell is the result of the difference between its formation and conversion into a different compound, i.e.,

**MET_LEVEL_=MET_FORMED_−MET_CONSUMED_** (1)

A cell requires an enormous diversity of processes to survive. It must be able to maintain cell metabolism and produce the building blocks that allow its growth and reproduction. In addition, some cells must synthesize very specialized compounds, such as osmoregulators, pigments and antibiotics, which require specific enzymes and cofactors available at the right levels [9]. Thus, cell metabolism has evolved to a highly complex network involving a massive number of metabolites and enzymes. The interactions between these enzymes and compounds can be structured in a series of reactions, or metabolic pathways, each one relating to particular cell requirements. Primary metabolic pathways are associated with catabolism (breakdown) and anabolism (synthesis), such as reactions to produce building blocks and free energy. Secondary metabolism pathways are associated with stress responses, such as pathway production of antibiotics or pigments [16,17].

**Figure 1:**
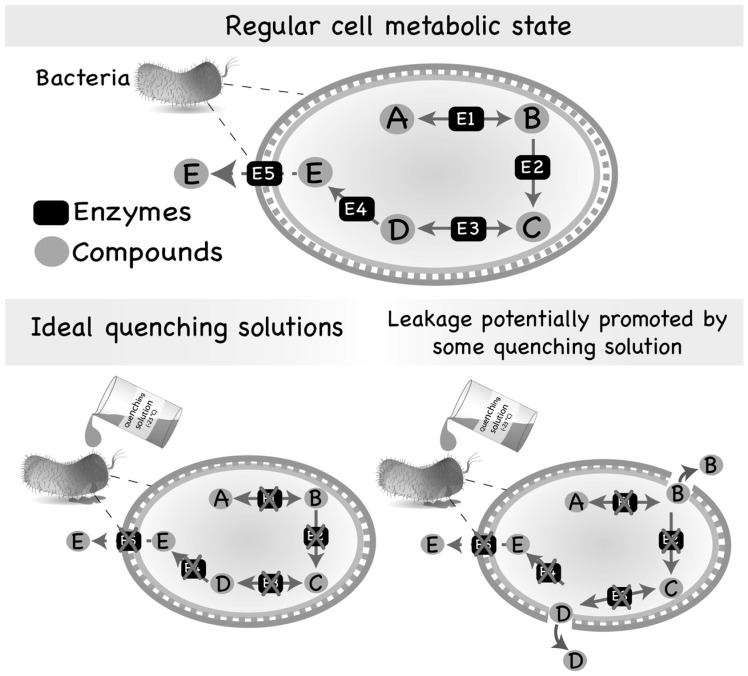
Regular metabolic state of a microbial cell and leakage promoted by quenching solutions. Most of the intermediates of the primary metabolism are usually substrates of numerous different enzymes and, consequently, are found in low abundance inside the cells. However, some intermediate metabolites might also be secreted by microorganisms if produced in adequate/abundant amounts. On the other hand, compounds of the secondary metabolism are generally substrates of few reactions and, thus, accumulate inside of the cell or are secreted to the extracellular medium. Quenching of microbial cells is generally performed by using an aqueous solution containing an organic solvent set to an extreme temperature (very hot or very cold) or an extreme pH (very acid or very basic). However, the quenching solution may interact with the cell envelope, damaging its structure and, consequently, producing pores through which intracellular metabolites can leak to the extracellular medium.

As primary metabolism is related to energy generation and cellular synthesis, its intermediates are mostly substrates and products of numerous different enzymes (Figure 1). These enzymes generally show a high rate of activity. Consequently, metabolites involved in primary metabolism have a particularly fast turnover rate within the cell and are very likely to be found at low abundances. Secondary metabolism pathways, on the other hand, are predominantly related to low growth rate, stress response and breakdown of cellular components [13]. When growth is limited, the secondary metabolism prevails. Intermediates from the secondary metabolism are precursors of only a small number of reactions and, therefore, have a considerably slower turnover rate when inside the cell. Consequently, the intracellular levels of intermediates from the secondary metabolism are generally higher than the intracellular levels of intermediates from the primary metabolism [9,18].

Metabolites present in the extracellular environment, on the other hand, are originally part of the medium composition, secreted by the cells, the product of cell lyses or the result of polymer degradation [19]. Enzymes, if not absent, are generally found in much lower abundances extracellularly and considerably diluted in the extracellular medium. Therefore, the turnover rate of metabolites present outside the cells is considerably lower when compared to the turnover rate intracellularly [20]. While factors such as metabolite interaction, temperature and light can change the levels of extracellular compounds, the main source of variability for these metabolites is the living cells in the medium. Thus, to avoid drastic changes in the extracellular pool of metabolites, the microbial cells must be quickly removed from the media and a quenching solution may also be used to stop potential degradation of metabolites [7,21]. It is also important that long exposure to light is avoided and samples are always kept at low temperatures (<20 °C) [9].

## 3. Microbial Cell Envelopes and Leakage of Intracellular Metabolites

Cells of microorganisms are encapsulated by a cell-wall matrix, which protects the cell from osmotic pressure while also providing it with both strength and shape. In this section we characterize the different microbial cell envelopes and discuss their importance on intracellular metabolite leakage during sampling and quenching.

### 3.1. The Microbial Cell Envelope

Although different microorganisms present different cell-wall matrixes, in most cases the cell wall is composed of lipids, polysaccharides, peptides and glycopeptides. Most bacterial cells present a thick or thin layer of peptidoglycan bounded by anionic polymers (e.g., teichoic acid) and a continuous layer of glycopeptides (*murein sacculus*) surrounded by phospholipids and lipopolysaccharides [22]. A technique called Gram staining is widely used to differentiate between two types of cell-wall matrix (e.g., Gram positive or negative bacteria). The Gram-staining technique is considered a basic procedure in the identification of bacterial cells and is routinely applied in microbiology [23].

The envelope of yeast cells differs from those of bacterial cells in that the former is composed of low amount of lipids, a higher percentage of proteins (30%–50% of the cell-wall matrix composition) and polysaccharides, such as glucans and mannans [24,25,26]. It is thicker than the bacterial cell wall and its thickness increases with age. Interestingly, yeasts’ cell walls also contain a small proportion (1%–2%) of chitin (polysaccharide), which is the main component of the exoskeleton of insects and crustaceans [25].

The envelope of filamentous fungi cells (moulds) is quite diverse and considerably different from the envelope of yeast and bacterial cells. Filamentous fungi are considered a unique group of organisms combining chitin and glucans in their cell-wall structure. In some species, chitin can represent 20% of the cell-wall composition while glucans may represent about 50%–60% of its structure. The rest of their cell-wall structure contains glycoproteins (15%–30%) and other components that vary considerably among species [26]. However, there are dimorphic fungal cells that alternate between yeast and hyphal stages depending on environmental conditions. 

Microalgae are very particular and also highly diverse in their cell envelope composition. Most of them, such as *Haematococcus pluvialis*, *Chlamydomonas monoica* and *Chlamydomonas reinhardtii*, exhibit a drastic change in the structure of their cell walls according to their growth phase. For example, most microalgae have an increase in resistant biopolymers, such as sporopollenin, as a component of their cell wall when entering the cyst stage [27,28]. In addition, some species, such as *C. reinhardtii*, have seven distinct layers composed of carbohydrates, glycoproteins and hydroxyproline-rich proteins [29]; and diatoms have silicon deposits in their cell walls [30]. The total wall thickness is quite variable among species, ranging from 100 nm to 440 nm, depending on growth conditions and growth phases [31].

Like microalgae, the compositions of a protozoa’s cell walls also vary considerably between species and are dependent on developmental stage. The cell-wall composition of *Acanthamoeba polyphaga*, for example, ranges from being completely comprised of cellulose to a combination of proteins and polysaccharides [32]. Because many of these microorganisms are parasites of human cells, most studies of their cell-wall structure focus on glycocalyx [33], the surface coat covering the cell-wall matrix, so that little is known about the proper cell-wall structure.

### 3.2. Leakage of Intracellular Metabolites

Most quenching methods applied to microbial cells are generally performed using different solvent solutions combined with extreme temperatures (very cold or very hot) or extreme pH [34,35]. However, these solutions may potentially interact with the constituents of the cell-wall matrix and damage its structure, resulting in the release of intracellular metabolites into the extracellular medium (Figure 1). As the volume of the extracellular medium is generally much larger than the intracellular volume of cells, these metabolites released into the medium become highly diluted and their detection is difficult by most of the analytical methods that are currently available. In addition, the presence of extracellular compounds interferes with the quantification of intracellular compounds, resulting in the wrong estimation of metabolite abundances [9,21].

Since different cell types have distinct cell-wall composition, the amount of intracellular metabolite leakage caused by different quenching solutions can differ between organisms. Some quenching methods may produce satisfactory results with one cell type but not another. Thus, the choice of best quenching method depends on the cell type under study.

## 4. Overview of Available Quenching Methods for Microbial Cultures

The need to quickly quench microbial cells’ metabolisms in order to obtain an accurate measurement of intracellular metabolites levels was recognized as early as 1960 [36]. Since then, it has been widely discussed by many biochemists and biologists [10,13,34,35,37,38,39,40,41,42,43,44,45,46,47,48,49,50]. One of the earliest methods proposed involved sampling the culture broth directly into an acidic solution of perchloric acid [34,44]. This approach was once considered very efficient; however, it has since been shown to have some disadvantages for metabolomics. The perchloric acid solution disrupts the cell envelope and releases intracellular metabolites into the extracellular medium, resulting in a single mixed pool of metabolites containing both intra- and extracellular compounds [38]. The quantification of intracellular compounds is then estimated by subtracting the level of extracellular compounds in the spent culture media. However, this approach generates large variability in the quantification of intracellular metabolites due to interference from highly concentrated media components during chemical analysis and chemical degradation of pH labile metabolites [51].

Saez and Lagunas (1976) addressed part of this when they developed a method able to quench the cell metabolism and extract intracellular metabolites in two different steps [47]. Using fast filtration followed by biomass immersion in liquid nitrogen, they were able to separate living cells and cultured media. The low temperature of liquid nitrogen slows down the cell metabolism and decreases the turnover of metabolites, providing extra time for the metabolite extraction. They subsequently performed intracellular metabolite extraction using acidic and alkaline solutions. However, this method requires more than 10 s per sample to actually quench cell metabolism and, consequently, it is unsuitable for analysing compounds of fast turnover rates such as ATP, NADH, pyruvate, glutamate, and many others [47]. 

In 1992, de Koning and van Dam proposed a quenching method that is until now considered the gold standard in quenching of microbial cells [49]. It uses direct sampling into 60% v/v methanol solution kept at −40 °C. The cell biomass is then separated from the culture medium by centrifugation at 3000 rpm for 20 min. The fast sampling strategy combined with the low temperature used by de Koning and van Dam arrests enzymatic activity of yeast cells in less than 1 s. The cells are then further submitted to intracellular metabolite extraction.

The use of cold-methanol solution has been considered very efficient and its use continues to be popular these days. However, an appropriate quenching method for microbial cultures must also prevent leakage of intracellular metabolites into the extracellular medium during quenching. Although de Koning and van Dam (1992) reported that the use of cold-methanol solution as a quenching agent promoted no, or very little, cell leakage [49], recent studies have shown that the membrane of yeast and bacterial cells are actually vulnerable to cold-methanol solution [10,13,18,21,40,41]. Bolten and co-workers (2007), for example, showed a 90% reduction in the concentration of free amino acids when quenching microbial cells with cold-methanol solution [13]. Variants of the original method proposed by de Koning and van Dam (1992) have been proposed for quenching yeast and bacterial cells [13,38,40,52,53]. Although some satisfactory results were observed, the use of cold-methanol solution for quenching the metabolism of microbial cells remains controversial.

Other quenching methods have been developed since the introduction of cold-methanol solution. In 2002, Chassagnole and co-workers used liquid nitrogen (−196 °C) for quenching cells of *Escherichia coli* [54]. However, because the liquid nitrogen freezes the biomass, it is very likely to produce ice crystals that can damage the cell membrane and promote leakage of intracellular metabolites. Thus, this method is not commonly used. However, Wittmann and co-workers (2004) and Bolten and co-workers (2007) proposed quenching bacterial cells by applying fast filtration under vacuum followed by biomass washing using either cold or room-temperature saline solution [13,41]. Although efficient for quenching reactions involving amino acids and some tricarboxylic acid intermediates, these methods take up to 45 s per sample if performed manually, which is not suitable for quenching metabolic reactions with fast turnover rates. 

Villas-Bôas and Bruheim (2007) [21] presented what they called “the promising quenching solution for accurate intracellular metabolite analysis of microbial cells”. They compared their novel method, based on a solution of cold glycerol–saline at −23 °C, to the well-known 60% v/v cold-methanol solution proposed originally by de Koning and van Dam and reported an excellent improvement in the recovery of intracellular compounds. Some metabolites, such as 3-hydroxyoctanoate, caprinate, glycerate, 2-oxoglutarate, pyroglutamate and dehydroabietate, were only detected in samples quenched by cold 60% v/v methanol solution and the abundances of all other intracellular metabolites were significantly higher (some of them more than 100-fold higher) than in samples quenched by cold glycerol–saline solution. However, the intracellular metabolite leakage associated with this method (if present) could not be directly quantified by the authors due to great interference of glycerol in the supernatant of quenched samples [55]. Nonetheless, based on the different levels of intracellular metabolites, the authors reported that using cold glycerol–saline solution as a quenching agent assured a much lower level of intracellular metabolite leakage when compared to cold-methanol solution. 

The great majority of the quenching methods developed to date target bacterial and/or yeast cells. Few quenching methods have been reported that halt the metabolism of filamentous fungi, microalgae and protozoa. As the cell-wall compositions within these groups of microorganisms are very diverse, developing quenching methods suitable for the range of different cell structures is very difficult. 

Table 1 summarises some literature resources for widely used and modified quenching protocols for microbial cells. We consider a combination of cold glycerol–saline solution and centrifugation followed by methanol–water solution to be the most efficient strategy for quenching cells. 

### 4.1. Bacterial Cells

The most cited methods for quenching bacterial cells are based on perchloric acid solution [34,44], cold-methanol solution [49], fast filtration combined with saline solution [13,41], glycerol–saline solution [21] and liquid nitrogen [47]. Some of these methods also consider using buffered solution in order to prevent large variation in pH. However, most aqueous solutions containing organic solvents may damage the cell-wall matrix and promote leakage of intracellular metabolites.

Analysts must be aware that bacterial cells are particularly sensitive to cold shock and that intracellular metabolites may leak when the cells are subjected to quick changes in temperature [41,53]. According to Leder (1972), the cold-shock phenomenon can be prevented or minimized by a simultaneous hyperosmotic transition [59]. Because the hyperosmolarity dries the cell’s periphery, it increases the interaction between the membrane lipids preserving the cell’s integrity. Others have also demonstrated that osmotic equilibrium between quenching solution and cell culture prevents cell leakage in Gram-positive and Gram-negative bacteria [60,61].

### 4.2. Yeast Cells

Yeast cells are considered less sensitive than bacterial cells against the cold-shock phenomenon and organic solvents. However, the traditional cold methanol–water solution [49], buffered and non-buffered, seems to promote cell leakage significantly when applied to yeast cells [10,62]. Villas-Boas and Bruheim (2007) compared the efficiencies of cold glycerol–saline solution, cold methanol–water solution, glycerol–water solution, glycerol–saline solution and glycerol–mannitol solutions in quenching yeast and bacterial cells [21]. They reported a considerable leakage promoted by the traditional cold-methanol solution and also reported that the cold glycerol–saline solution produces better results in recovering intracellular metabolites. In 2008, Canelas and co-workers once again reported the leakage promoted by the traditional cold methanol–water solution and proposed the use of 100% cold-methanol solution, which seems to produce considerably less leakage of intracellular compounds [10]. 

Other methods (Table 1) such as boiling ethanol, perchloric acid and liquid nitrogen have also been tested for quenching yeast cells; however, none of them seem to prevent leakage of intracellular compounds and some of these methods are particularly difficult to apply.

### 4.3. Filamentous Fungi and Bacteria

Quenching filamentous fungi and filamentous bacteria is a more complex process than quenching bacterial or yeast cells. The cell structure of filamentous organisms is very diverse. In addition, cultures of filamentous fungi are generally more viscous and heterogeneous than cultures of unicellular microorganisms. Consequently, there is not yet a standard method for quenching all types of filamentous fungi or bacteria. 

Some methods propose the use of liquid nitrogen [17], cold-methanol solution [48] and fast filtration combined with cold-methanol solution [58]. Liquid nitrogen is efficient for stopping the cell metabolism but prevents further separation of intra- and extracellular metabolites if cultures are grown in liquid broth. Ruijter and Visser applied 60% v/v methanol buffered with 200 mM tri-ethanolamine at −45 °C for quenching the metabolism of *Aspergilus niger* [48]. Based on the quantification of metabolites, such as ATP, ADP and AMP, the authors reported no significant leakage of these targeted intracellular metabolites to the extracellular medium. Further biomass separation was performed by vacuum filtration after quenching. However, there was no comprehensive evaluation of intracellular metabolite leakage apart from those targeted phosphorylated nucleotides. Fast filtration combined with cold-methanol solution was also reported as efficient in quenching the cell metabolism of filamentous fungi, and it also allows the separation of intra- and extracellular metabolites [58]. However, fast filtration takes up to 45 s per sample, which may result in high variability in the estimation of intracellular metabolites levels with high turnover rates.

### 4.4. Protozoa

As with filamentous fungi, there are very few protocols describing quenching methods for protozoa cells. Protozoa are also a very diverse group of microorganisms in regard to their cell structure at different life phases. De Souza and co-workers (2006) suggested the immersion of the whole culture broth into a bath with dry ice–ethanol until the cell suspension reached 0 °C in order to quench cells of the protozoa *Leishmania donovani* in promastigote life stage. Afterwards, a solution of cold phosphate-buffered saline (0 °C) was used to wash the cells and separate the biomass from the rich culture medium. The authors monitored the presence of intracellular metabolites in the supernatant and concluded that this quenching method induced none or very little leakage of intracellular metabolites [57].

### 4.5. Microalgae

As with filamentous fungi and protozoa, the cell envelope of microalgae is also diverse and may be very complex according to the developmental stage. Bolling and Fiehn (2005) modified the quenching method developed by de Koning and van Dam (1992) for quenching the metabolism of the microalgae *Chlamydomonas reinhardtii* [56]. They sprayed a cell suspension of *C. reinhardtii* into a solution of 32.5% methanol–water supplied with tris-acetate phosphate (TAP) macro salts. The authors used TAP medium in presence of labelled [U^−14^C] acetic acid in order to evaluate the quenching efficiency of this method, which proved to be very satisfactory. Other methods may be modified and adapted for quenching the cells of different species.

## 5. Devices for Fast Sampling

Many factors may influence the efficiency of a quenching method. Within these, the time between sampling and the actual quench of the cell metabolism is considered one of the most important. Because the inter-conversion of metabolites may happen in the order of seconds, a quick sampling and quenching method is essential for producing an accurate picture of the in vivo cell metabolism. Recently, a direct sampling method for the real-time metabolome profiling of bacterial cells (e.g., *E. coli*) using a high-resolution mass spectrometer (time of flight) has been published by Link et al. [63]. This method proved to be superior in comparison to manual sampling, thus allowing the monitoring of the dynamics of metabolic activity across the full mass range on a resolution of 15–30 s in different organisms [63]. Although it is indeed a better method for metabolome profiling, such a high-resolution mass spectrometer is still not available for most of the researchers in the metabolomics community. Therefore, quick sampling of microbial cultures either from culture flasks or from bioreactors is used by many, and some examples and advancements are presented below.

### 5.1. Sampling from Culture Flasks

Culture flasks are usually sampled manually. Generally, pipettes or syringes are used to harvest a specific amount of sample and quickly spray it into a flask containing the quenching solution. Therefore, the analyst must be very well trained in rapidly transferring reproducible amounts of culture broth to quenching flasks. A good alternative is the use of a syringe prefilled with the quenching solution [13], which reduces the time frame required for the quenching solution to mix with the living cells. Alternatively, the analyst can pour the biological sample directly into quenching solution and quickly homogenize it, as demonstrated in an online video [58].

Independent of the method used, it is important to guarantee fast sampling and a controlled amount of sample harvested each time in order to minimise technical variability. For that, the analyst can always weigh the flask containing the quenching solution before and after sampling.

### 5.2. Sampling from Bioreactors

Manual, semi-automated and fully automated techniques have been developed for sampling microbial cultures from bioreactors. Manually, syringes are the most common technique used for the quick harvesting of microbial culture from bioreactors. However, even experienced analysts are unable to manually sample bioreactors within 4 to 5 s/sample. Therefore, several semi-automated and fully automated sampling devices have been developed to improve sampling time and achieve better reproducibility. 

Semi-automated devices were presented by Harrison and Maitra (1969) [34] and afterwards improved by Theobald et al. (1993) [64]. This device had specially designed valves to aseptically harvest and quench biological samples in about 0.5 s per sample. However, some of the steps required by these methods were still performed manually and, thus, their reproducibility depended on the skills of the analyst operating the device. In 1996, Larsson and Törnkvist addressed part of this problem when they developed an electronically controlled valve [65]. An electronic timer was used to set a specific time frame for the valve to be opened and release the biological sample directly into a tube containing the quenching solution. The authors validated this method by quantifying glucose consumption in fed-batch cultures of *Escherichia coli* and *Saccharomyces cerevisiae*. However, this system still depended on an analyst to insert and collect the testing tubes, which is time-consuming and precludes high-frequency sampling. 

It was not until 1998 that the first fully-automated system for harvesting and quenching microbial cells was built [66]. Schaefer and co-workers (1999) developed this device to dynamically investigate the glucose metabolism of *E. coli*. The main idea consisted of continuously spraying culture broth samples into sample tubes filled with quenching solution that moved at a defined speed underneath the bioreactor. The authors used a magnetic pinch valve inserted in the bottom of the bioreactor pointing to a magazine located underneath. This magazine had the capacity to horizontally transport 16 quenching tubes. As a result of the higher pressure inside the bioreactor, when the magnetic valve was opened, it continuously sampled culture broth at 3.3 ml s^−1^ into each individual sample tubes containing cold quenching solution moving underneath the bioreactor. This system allowed a sampling rate of 4.5 samples per second. Many other systems have been developed using similar technology [67,68,69]. Each of these systems has advantages and drawbacks when applied to different experimental conditions. Thus, we suggest a careful analysis of the sampling system before applying any of these technologies.

#### Stopped-Flow Systems

When studying the dynamics of the cell metabolism, a commonly applied strategy is called stimulus-response or continuous perturbation experiments [49,64,66]. It consists of quickly disturbing the cell metabolic state growing in chemostats and recording the concentration of metabolites at different time points. The different levels of each metabolite at each time point allow the analyst to infer reaction rates and, consequently, understand the regulation of specific pathways. Stimulus response is achieved by, for example, applying a substrate pulse to a substrate-limited culture under steady state [16,70]. For instance, a concentrated solution of glucose can be injected into a glucose-limited chemostat culture to study the regulation of glycolysis. The solution of glucose shifts the cell metabolic state, giving the possibility of recording the gradual changes in metabolite concentrations through the different reactions of the pathway. 

Stimulus-response experiments can be performed using the fast sampling systems described above. However, it has some drawbacks when multiple stimuli are to be studied. Every stimulus must be applied when the cell culture reaches the steady-state condition. However, when the first perturbing agent is introduced into the cell culture, the culture steady-state is lost and, depending on the organism under study and the dilution rate applied, it may require more than 48 h and many litres of media to reach a new steady-state. In addition, there is a time frame required for the perturbing solution to mix with the cell culture and actually affect the metabolism of all living cells in the bioreactor. This time frame depends on the stirring speed, the volume of the culture and the viscosity of the liquid. Finally, the high volume of the bioreactors requires a large amount of perturbing agent, which may increase significantly the overall costs of the experiment. 

A solution for these limitations is presented by the sampling systems called stopped flow. These systems rely on applying the desired perturbations in a secondary flask, or outlet, sitting outside the bioreactor. A defined volume of cell culture is driven to the outlet where the stimulus solution is simultaneously injected. The low volume of the secondary flask promotes a quick and uniform mix between the stimulus solution and the cell culture. In addition, as the whole pulse-response process happens outside the bioreactor, the steady-state is maintained and a new experiment can be subsequently performed in little time. An efficient stopped-flow sampling system must be able to sample and quench microbial cells at a high frequency and in a reproducible manner.

Many different stopped-flow systems have been developed to date. In 1992, de Koning and van Dam proposed a stopped-flow system using a freeze quench device in order to analyze changes in glycolytic metabolites [49]. However, this system was not connected to a continuous growth culture, which avoided the analysis of the glycolytic pathway starting from a defined physiological condition or steady-state condition. In 2002, Buziol and co-workers modified the method developed by de Koning and van Dam. They built a fully-automated stopped-flow sampling system connected to a standard port of the bioreactor [71]. This system was built using a mixing chamber located outside the bioreactor and five three-way valves controlled by computer software. A sample of the culture broth was mixed with the disturbing agent in the mixing chamber and redirected to a sequence of sample tubes located at distinct distances from the bioreactor. The different distances travelled by the sample represent the gradual changes in the metabolic state of the cell. This system also allows individual sample volumes and the first sample is harvested in less than 100 ms after the injection of the disturbing solution. However, there is still one limitation. This system did not allow the exchange of oxygen while the sample was processed outside the bioreactor. Consequently, the metabolic state of the cell culture was also influenced by oxygen limitation when aerobic experiments were performed.

Visser et al. developed a stopped-flow sampling system that overcame this problem [37]. The so-called BioScope was built using a flow channel with oxygen-permeable silicon tubing, which allowed oxygen exchange even when the culture broth was being directed to 11 different sample tubes located outside of the bioreactor. In addition, its silicon tubing was built using a serpentine configuration, which improved the mixing rate between the culture and the perturbing solution. Later this system was further improved [72] by the use of a new technology of O_2_/CO_2_ silicon membrane, which is more flexible for switching from aerobic to anaerobic conditions and requires lower to minimum maintenance.

## 6. Extraction of Intracellular Metabolites: Disruption Methods for Microbial Cell Envelopes

Different factors are directly responsible for the shape and strength of the cell envelope (structure and composition) of different microorganisms. The complexity of a microbial cell envelope mainly depends on the structural polymeric composition of the cell and the degree of cross-linking between these polymers and other cell-wall components. Therefore, the major resistance that needs to be overcome during the disruption of cell envelopes is the covalent chemical bonds between the structural components. Both mechanical and non-mechanical methods are widely used for cell envelope disruption [9]. Table 2 summarizes the different extraction protocols used by the metabolomics community for the extraction of the intracellular metabolites from microbial samples. 

Mechanical disruption of the cell envelope can be affected by different factors, such as polymer concentration within the cell wall, cell size and shape and degree of cross-linking between the polymers. However, the ease of mechanical cell disruption depends on the complexity and composition of the cell envelope. For example, the cell wall of Gram-negative bacteria can be easily ruptured using mechanical methods when compared with the cell wall of Gram-positive bacteria. Similarly, the cell-wall composition of yeasts and filamentous fungi is more complex than that of bacteria; therefore, the disruption of their cell wall requires stronger mechanical forces [9]. Different mechanical cell-disruption methods are available (Table 2). Most of the methods are not very popular in the preparation of microbial metabolome samples as they result in the release of small and large metabolites, which is not desired in most metabolomics studies. 

Non-mechanical cell enveloped disruption methods are generally preferred for the extraction of the intracellular metabolites from microorganisms. In this case, chemical or physical agents are used to make the cell envelope permeable so that the intracellular metabolites can be released into the cytoplasmic medium. Different disrupting agents, such as, enzymatic, mechanical and chemical, can be used for the preparation of intracellular samples. The application of both enzymatic and physical agents is quite limited in metabolomics because of their ability to degrade the polymeric components of the cell envelope and result in leakage of large molecules. Some of these enzymatic-physical methods can be combined with chemical methods to enhance the extraction process. Chemical lysis of the cell envelopes is part of the majority of protocols developed to extract intracellular metabolites from microbial cells (Table 2). Ideally, these protocols should be modified to obtain optimum performance based on the cell-wall structure and composition of microorganisms [9]. The next sections of this chapter will be focused on the methods that are commonly used to extract the intracellular metabolites from microorganisms. 

### 6.1. The Extraction of Intracellular Metabolites by Chemical Lysis 

The most popular methods for intracellular metabolite extraction are based on the application of chemical agents (Table 2). Metabolites are generally distributed between two phases according to their partitioning coefficients, solubility, solvent temperature, and the relative volumes of the phases. The aim in this case is to concentrate metabolites in a single phase, which can be achieved by using chemical agents [9]. While selecting an appropriate chemical agent, one should consider the metabolite extraction rates associated with this specific chemical. The extraction rates may change in response to temperature and diffusion rates in the two phases that would allow the solvent to get into the cell envelope to extract the intracellular metabolites. As a result, the extraction rate of a chemical agent is also directly linked to the degree of cell permeabilization. Therefore, the choice of chemical agents and extraction conditions depends on the type of microbial cells targeted and the groups of metabolites of interest. Many chemical extraction methods simply aim at extracting a few metabolite species (e.g., fatty acids, amino acids). However, the ideal metabolome analysis aims at extracting as many classes and species of metabolites as possible. Hence, the metabolomics community has been emphasizing the necessity to use, when applicable, multiple extraction methods in order to obtain a comprehensive as possible intracellular metabolite profile [12,73,74,75].

Both polar (e.g., methanol or ethanol) and non-polar (e.g., ethyl acetate, hexane and chloroform) solvents are extensively employed for the extraction of microbial intracellular metabolites. The organic solvents have the ability to weaken the cell wall, cell membrane proteins and lipids; therefore, they can form pores in the cell envelope. Then the intracellular metabolites are released through the pore and extracted into the organic solvent. However, the ideal solvent-extraction methods should require a small of amount of solvent. In addition, the volume of sample, the extraction time and the broadness of the coverage metabolites are three other important parameters while choosing a suitable extraction protocol [9,18,73]. In this section, we are going to discuss only the most popular extraction protocols that are commonly used to prepare the intracellular samples from microorganisms. 

#### 6.1.1. Boiling Ethanol

The use of buffered boiling ethanol (75% v/v) is a simple and rapid intracellular metabolite extraction protocol. This method was first reported by Entian et al. [94] and later it was further modified and improved by Gonzalez, Francois and Renaud [40] for the extraction of polar metabolites from yeast cells. In this method, the quenched microbial cells are exposed to buffered boiled ethanol (80 °C) for several minutes, which causes the deactivation of enzymes and proteins. The heating also enhances cell disruption, thus allowing the extraction of water-soluble intracellular metabolites. After that, the ethanol–water mixture is evaporated and the pellets are resuspended in water prior to analysis. One of the main advantages of this method is its good reproducibility [9]. However, several authors reported that there was poor recovery for several classes of metabolites, such as, phosphorylated metabolites, nucleotides and tricarboxylic acids [18,51]. Furthermore, this extraction protocol is not suitable for thermo-labile metabolites and there is a chance of oxidation for reduced metabolites [9]. However, the boiling ethanol extraction method is one of the most popular methods that has been used for the extraction of intracellular metabolites for many years. 

#### 6.1.2. Cold Methanol

Cold-methanol extraction is an extensively used method that is also another simple and fast method for the extraction of the intracellular metabolites from a wide range of microbial cells. For instance, cold methanol has been used to extract metabolites from bacteria [51,73,74], yeasts [18,73,75,80] and filamentous fungi [17,73]. This is a very powerful method that uses only a single organic solvent that can be easily removed from the samples simply by sample evaporation. Moreover, the extraction process is generally performed under very low temperature (<−20 °C), thus it is suitable for thermo-labile metabolites. The main disadvantage of this method is the lack of complete enzyme inactivation and, thus, there is a risk of change on intracellular metabolite pools. This method also showed excellent reproducibility and great recovery for polar and mid-polar metabolites. However, the recovery of non-polar metabolites is not as good as polar metabolites [18]. Cold-methanol extraction is sometimes coupled with freeze–thaw cycles or sonication in order to enhance the cell permeability [73].

#### 6.1.3. Buffered Methanol–Chloroform–Water

The extraction of intracellular metabolites (total lipids) from animal tissue using buffered methanol–chloroform–water was first reported by Folch et al. [95]. However, the first method that was employed for a microbial system was published by de Koning and van Dam [49]. They used a mixture of buffered methanol–water–chloroform at low temperature (*−*40 to −20 °C) while shaking the mixture vigorously (about 300 g for 45 min) to extract polar metabolites of yeast cells. This method is highly useful for the extraction of both polar and non-polar metabolites from bacteria, yeasts and filamentous fungi. Thermo-labile metabolites can also be extracted by this method as it is performed at a low temperature. Even though chloroform is known as a toxic and carcinogenic agent, it helps to denature all the enzymes within microbes and stops further chemical reactions. However, appropriate precautions need to be undertaken while using this method to avoid the hazardous effects of chloroform. In addition, it is a laborious and time-consuming method, and the buffers also may cause problems for different analytical techniques [9]. However, a very good recovery of phosphorylated and thermo-labile compounds was obtained from this method [18]. 

#### 6.1.4. Hot Water

Hot water has been used for the extraction of bacterial amino acids since 1950s [96]. Later on, some other researchers also applied this technique for the extraction microbial metabolites, but poor recovery was obtained for the targeted metabolites [97]. However, Hiller, Franco-Lara and Weuster-Botz [82] published a detailed protocol where they achieved excellent recovery and reproducibility for the intracellular metabolites from *E. coli* using pre-heated de-ionized hot water (95 °C, 5 min). This method is advantageous because it is very simple and easy to perform. In addition, enzyme activity is more likely to stop because of the high temperature of the water. However, this method can only extract polar metabolites and is not suitable for thermo-labile metabolites. 

#### 6.1.5. Acidic Extraction

The extraction of intracellular metabolites using acids (e.g., perchloric acid, trichloroacetic acid and hydrochloric acid) is one the classical methods that has been used for many years. This method is proved to be a good one for polar and acid-stable metabolites and has been used for the extraction of intracellular metabolites from bacteria, yeasts and filamentous fungi [17,18,38,74,84]. The extraction process is usually performed at a low temperature (0–4 °C) and a freeze-thaw cycle is also generally used to enhance the extraction process. However, the pH of the sample needs be neutralized after extraction is completed. The acidic extraction method showed excellent recovery of amines and polyamines, but poor recovery was reported for other metabolites. Moreover, the reduced metabolites might be oxidized during the extraction and there might be hydrolysis of proteins and polymers [9].

#### 6.1.6. Alkaline Extraction

Alkalis are mainly used for the extraction of intracellular metabolites from yeast and filamentous fungi. This extraction process is also carried out at a low temperature (0–4 °C) coupled with a freeze–thaw cycle. This is an excellent method to disrupt the microbial cell wall that deactivates enzymes promptly at extreme high pH. However, there are a few drawbacks to this method, which include poor recovery of intracellular metabolites, saponification of lipids and hydrolysis of proteins and polymers [18,51]. Once again, a neutralization step is required to adjust the pH, which causes the production of salts that are removed from the sample by centrifugation [9]. 

### 6.2. Mechanical Disruption of Cell Walls

Although many mechanical cell-disruption protocols (e.g., ultrasonics, microwave, French press and grinding) are widely used to extract the metabolites from plant and animal cells, these are not preferred methods for the extraction of microbial intracellular metabolites. However, two methods, such as supercritical fluid extraction (SFE) and pressurized liquid extraction (PLE) have been used for the extraction of intracellular metabolites from some microorganisms. We will briefly discuss their application to microbial systems in following sub-sections.

#### 6.2.1. Supercritical Fluid Extraction (SFE)

SFE allows the extraction of non-polar to mid-polar compounds from bacteria, yeast and filamentous fungi [86]. This method usually makes use of carbon dioxide as a supercritical fluid for the extraction of intracellular metabolites. Sometimes, nitrous oxide and xenon are also used. Moreover, methanol or ethanol is also added in addition to carbon dioxide as a modifier so that polar compounds can also be extracted from the microorganisms [88]. This is a fast method that requires a small amount of solvents and samples. In addition, this method can be automated and coupled to analytical instruments, such as gas chromatograph and mass spectrometer. However, as SFE works under high pressure, labile metabolites might be decomposed. 

#### 6.2.2. Pressurized Liquid Extraction (PLE)

PLE is mainly used for the extraction of secondary metabolites produced by the microorganisms. However, this method has not been used by many researchers in the field of metabolomics. But this method is suitable for high-throughput screening and very concentrated metabolite extracts can be obtained [91]. On the other hand, only thermostable metabolites can be extracted using PLE [9].

## 7. Conclusions

Quenching is certainly one of the most crucial steps in metabolomics studies. An efficient quenching method for microbial cultures must be fast, reproducible, and it must prevent leakage of intracellular metabolites to the extracellular medium. Several quenching methods have been developed to date; however, each method shows a considerable specificity to the organism under study and the culture medium in use (e.g., liquid medium, agar plates or natural substrates). Recently, very efficient fully automated systems have been developed to increase the sampling speed and reduce the variability introduced by human errors. However, they are mostly purpose-made and not commercially available. There is a need for more robust quenching methods and also more accessible equipment, which may be achieved by merging the knowledge of specialists from distinct fields, such as biology, chemistry and engineering. 

The standardization of analytical protocols and extraction methods is the most discussed topic among members of the metabololomics community. These discussions over the last decade make it clear that no single analytical technique is sufficient to determine the comprehensive metabolite profiles from biological samples; rather, a combination of different techniques has been suggested for acquiring as much information as possible. Similarly, recent work from different laboratories also raises general consciousness about the necessity of a global (and standard) extraction protocol that can be used to extract as many metabolites as possible. If that is not realistic or not achievable, then the combination of extraction methods by using solvents with different polarities would be very useful for obtaining global and more accurate intracellular metabolite profiles from microorganisms. In this way, we would be able to achieve a more precise biological interpretation of the metabolomics data.

## Figures and Tables

**Table 1:** Summary of literature reports on widely used methods for quenching microbial cultures.

Year	Quenching Method	Organism	Reference
1963	Perchloric acid solution	Bacteria (*Aerobacter aerogenes*)	[44]
1976	Fast filtration followed by liquid nitrogen immersion of biomass	Yeast	[47]
1992	Cold-methanol (60% v/v) solution	Yeast (*Saccharomyces cerevisiae*)	[49]
1996	Buffered methanol (60% v/v) solution at −45 °C	Filamentous fungi (*Aspergillus niger*)	[48]
1998	Dropping mycelium cultures in liquid nitrogen or spraying the culture on a cold methanol (60% v/v) solution followed by rapid centrifugation	Filamentous fungi (*Monascus ruber*)	[17]
2004	Quick filtration	Bacterium (*Corynebacterium glutamicum*)	[41]
2005	32.5% methanol solution in water supplemented with CaCl_2_, MgCl_2_ and KCl	Microalgae (*Chlamydomonas reinhardtii*)	[56]
2006	Immersion of culture flasks to ethanol–dry ice bath	Protozoa (*Leishmania donovani*)	[57]
2007	60% v/v cold-methanol solution with different additives	Bacteria (*Lactobacillus plantarum*)	[38]
2007	Fast filtration	Bacteria (*Bacillus subtilis*, *Corynebacterium glutamicum*, *Escherichia coli*, *Gluconobacter oxydans*, *Pseudomonas putida*, *and Zymononas mobilis*)	[13]
2007	Cold glycerol–saline solution	Bacteria and yeast (*Pseudomonas fluorescens*, *Streptomyces coelicolor* and *Saccharomyces cerevisiae*)	[21]
2008	Pure methanol at −40 °C	Yeast (*Saccharomyces cerevisiae*)	[10]
2010	Cold glycerol solution and fast filtration	Bacteria, yeast and filamentous fungi	[58]
2011	Comparison of four different quenching method based on aqueous cold-methanol solution	Yeast (*Pichia pastoris*)	[52]
2012	40% v/v of methanol solution at −20 °C	Mould (*Penicillium chrysogenum*)	[39]
2014	Automated fast filtration and on-filter quenching	Bacteria (*Escherichia coli*)	[11]

**Table 2:** Literature evidence of the application of different extraction methods for intracellular metabolites from microorganisms.

Extraction Method	Extracted Metabolites	Microorganisms	References
CHEMICAL EXTRACTIONS			
Boiling ethanol	Polar (thermostable)	*Sacharomyces cerevisiae*, *Aspergillus* sp., *Penicillium chrysogenum*, *Monascus ruber*, *Klebsiella oxytoca*, *Escherichia coli*, *Enterococcus faecalis and Lactobacillus plantarum*	[12,17,18,37,38,40,51,73,74,75,76,77,78,79]
Cold methanol	Polar and mid polar	*Sacharomyces cerevisiae*, *Aspergillus* sp., *Zymomonas mobilis*, *Penicillium* sp., *Klebsiella oxytoca*, *Bacillus subtilis*, *Pseudomonas putida*, *Gluconobacter oxydans*, *Corynebacterium glutamicum*, *Escherichia coli*, *Enterococcus faecalis and Lactobacillus plantarum*	[13,18,39,51,73,75,80,81]
Buffered methanol–water–chloroform	Polar and non-polar	*Sacharomyces cerevisiae*, *Escherichia coli*, *Bacillus licheniformis and Klebsiella oxytoca*	[12,18,51,74,81,82]
Hot water	Polar (thermostable)	*Sacharomyces cerevisiae*, *Escherichia coli and Klebsiella oxytoca*	[12,74,83]
Acidic extraction	Polar and acid stable	*Monascus ruber*, *Sacharomyces cerevisiae*, *Aspergillus niger*, *Klebsiella oxytoca*, *Bacillus licheniformis and Escherichia coli*	[17,18,38,74,78,81,84]
Alkaline extraction	Polar and alkali stable	*Monascus ruber*, *Sacharomyces cerevisiae*, *Aspergillus niger*, *Klebsiella oxytoca and Escherichia coli*	[17,18,51,74,76,78]
MECHANICAL EXTRACTIONS			
Superficial fluid extraction	Non-polar to mid polar	*Agaricus* sp., Gram-positive and Gram-negative bacteria	[85,86,87,88,89,90]
Pressurised liquid extraction	Secondary metabolites	Yeasts and microalgae	[89,91,92]
Microwave	Thermostable metabolites	Yeasts	[91]
COMBINATION OF CHEMICAL AND MECHANICAL EXTRACTION			
Pure cold methanol coupled to sonication	Polar, mid polar and stable	*Sacharomyces cerevisiae*, *Aspergillus* sp*.*, *Escherichia coli and Enterococcus faecalis*	[73]
Methanol and bead mill	Polar and mid polar	Clinically relevant bacteria	[93]
Cold methanol–water solution coupled to freeze–thaw cycles	Polar and mid polar	*S. cerevisiae*, *Aspergillus* sp., *Escherichia coli and Enterococcus faecalis*	[73]
